# Adjusting Behavioural Surveillance and Assessing Disparities in the Impact of COVID-19 on Gay and Bisexual Men’s HIV-Related Behaviour in Australia

**DOI:** 10.1007/s10461-022-03788-1

**Published:** 2022-07-27

**Authors:** Martin Holt, Curtis Chan, Timothy R. Broady, Limin Mao, James MacGibbon, John Rule, Ben Wilcock, Garrett Prestage, Benjamin R. Bavinton

**Affiliations:** 1 Centre for Social Research in Health, UNSW Sydney, Sydney, NSW 2052 Australia; 2 The Kirby Institute, UNSW Sydney, Sydney, Australia; 3 National Association of People with HIV Australia, Sydney, Australia; 4 Australian Federation of AIDS Organisations, Sydney, Australia

**Keywords:** Australia, COVID-19, HIV prevention, Men who have sex with men, Sexual behavior

## Abstract

COVID-19 has disrupted sexual behaviour and access to health systems. We adapted regular HIV behavioural surveillance of gay and bisexual men (GBM) in Australia in response to COVID-19, assessed the impact on the profile of the sample, the participants’ HIV-related behaviour, and whether COVID-19 may have accentuated existing disparities in the Australian HIV epidemic. Data collected from five states during July 2017–June 2021 were included (N = 31,460). The emphasis on online recruitment after COVID-19 led to smaller sample sizes, greater geographic reach, and a higher proportion of bisexual-identifying participants. Most participants (88.1%) reported physical distancing and 52.1% had fewer sex partners due to COVID-19. In the COVID-19-affected rounds (July 2020–June 2021), the number of male partners, recent HIV testing and pre-exposure prophylaxis (PrEP) use all fell, and HIV risk among the smaller group of participants who reported casual sex increased. COVID-related changes were generally more pronounced among GBM aged under 25 years, participants from suburbs with fewer gay residents, and bisexual men. These groups should be prioritised when encouraging GBM to reengage with HIV testing services and effective prevention methods, like condoms and PrEP.

## Resumen

COVID-19 ha interrumpido el comportamiento sexual y el acceso a los sistemas de salud. Adaptamos la vigilancia regular del comportamiento de hombres homosexuales y bisexuales (GBM) hacia el VIH en Australia en respuesta a COVID-19, evaluamos el impacto en el perfil de la muestra, el comportamiento relacionado con el VIH de los participantes y si COVID-19 puede haber acentuado las existentes disparidades en la epidemia australiana de VIH. Se incluyeron los datos recopilados de cinco estados entre julio de 2017 y junio de 2021 (N = 31 460). El énfasis en el reclutamiento en línea después de COVID-19 resulto en tamaños de muestra más pequeños, mayor alcance geográfico y una mayor proporción de participantes que se identifican como bisexuales. La mayoría de los participantes (88,1%) describieron participando en el distanciamiento físico y el 52,1% tuvo menos parejas sexuales debido a la COVID-19. En las rondas afectadas por COVID-19 (julio de 2020 a junio de 2021), disminuyó la cantidad de parejas masculinas, el uso reciente de pruebas de VIH y de la profilaxis previa a la exposición (PrEP), y el riesgo de VIH entre el grupo más pequeño de participantes que participaron en sexo casual aumentó. Los cambios relacionados con COVID fueron generalmente más pronunciados entre GBM menores de 25 años, participantes de suburbios con menos residentes homosexuales y hombres bisexuales. Se debe priorizar a estos grupos al alentar a GBM a volver a comprometerse con los servicios de pruebas de VIH y métodos de prevención efectivos, como condones y PrEP.

## Introduction

As well as inflicting significant levels of morbidity and mortality, the COVID-19 pandemic has caused widespread disruption to health systems, including the delivery of care for people with existing health conditions. Many research and surveillance activities have had to be delayed or adjusted to comply with COVID-19-related restrictions, potentially impeding the monitoring of and response to existing epidemics such as HIV [1, 2].

Regular behavioural surveillance of HIV-affected populations is recommended to assess behaviours that may increase the risk of HIV, the adoption of protective practices, and engagement with health services and programs, like HIV testing and treatment [3, 4]. Behavioural surveillance can function as an early warning system, identifying changes in practice that may lead to increases in HIV incidence, and as an evaluation tool, assessing the effectiveness of HIV programs in encouraging practices like condom use or pre-exposure prophylaxis (PrEP) uptake [5, 6]. It may also be used retrospectively, to try to explain how changes in behaviour have affected HIV diagnoses. A key challenge for behavioural surveillance systems is to remain responsive to changes in practice in affected populations, while maintaining sufficient continuity in the system so that key indicators and trends can be reported over time [6].

Australia is one of the few countries to have maintained annual behavioural surveillance of its main HIV-affected population, gay and bisexual men (GBM), for 25 years [6–8]. In the 5 years preceding the emergence of COVID-19, the focus of Australian behavioural surveillance was adjusting the system to guide and assess the adoption of new prevention strategies like PrEP and treatment as prevention [6, 9]. In 2019, for example, PrEP overtook condoms as the most commonly used HIV prevention strategy by GBM in Australia [10], associated with the first major declines in annual HIV infections for over 15 years [11–13]. However, the rollout of new prevention technologies has been uneven, with concerns raised that some groups remain at increased risk of HIV, due to lower levels of engagement with or difficulty accessing HIV services, including testing and PrEP. These include younger men, overseas-born GBM (particularly from Asia), those living outside inner-city, gay-friendly suburbs, and bisexual men [10, 11, 14–19]. Others have observed that the impacts of COVID-19 have been accentuated by racial and socioeconomic disparities [20–22]. It is therefore likely that the disruption caused by COVID-19 may have accentuated existing HIV-related disparities in Australia.

Australia began imposing COVID-19 restrictions in February 2020. In most states and territories, an initial ‘lockdown’ period was imposed in March 2020, recommending that people stay at home where possible, practise physical distancing and enhanced hand hygiene [23]. Many businesses and public venues were closed, and limits imposed on congregating inside or in public. Throughout 2020, restrictions varied by jurisdiction, generally easing in states/territories with few COVID-19 cases, and reimposed in states where community outbreaks were detected. Many states/territories achieved long periods without any COVID-19 cases detected, other than in compulsory hotel quarantine for international travellers. However, in the second half of 2021, outbreaks and lockdowns occurred in multiple states and territories, attributed to more infectious variants of COVID-19. To put this in context, as of July 2021 Australia had only recorded 33,908 COVID-19 cases, but this increased to 395,385 cases by the end of December 2021 [24].

Cohort and clinical studies observed the impact of COVID-19 on Australian GBM during 2020, noting dramatic declines in the number of sexual partners, HIV testing and PrEP use, particularly during the first 6 months of the pandemic, and when lockdown restrictions were (re)imposed [23, 25–27]. These changes were attributed to concern about acquiring or passing on COVID-19, avoiding unnecessary attendance at health services, and reduced social and sexual contact with other people. Similar patterns of reduced sexual activity and engagement with HIV testing and prevention were found in many other countries [21, 28–32], with some notable exceptions [33, 34]. Some studies found that reduced social and sexual contact were associated with stress, anxiety and feelings of isolation [28, 29, 35, 36], while others found that disparities (such as those related to age or ethnicity) accentuated economic impacts of COVID-19 and reduced access to HIV services [21, 22].

Here we document the way in which we adjusted HIV behavioural surveillance of gay and bisexual men to maintain ongoing monitoring and evaluation, but also to assess the impact of COVID-19 on GBM and the national HIV epidemic. We consider changes to sampling and key behavioural indicators, and whether COVID-19 has accentuated disparities in HIV testing, prevention and HIV risk. We discuss the implications of COVID-19 for the HIV response in Australia and the resilience of the behavioural surveillance system.

## Methods

### Participants and Procedures

Australia’s main behavioural surveillance system for HIV, the Gay Community Periodic Surveys, involves repeated, cross-sectional surveys of GBM at venues, events and online [6, 7]. They have been conducted since 1996 and involve recruitment in seven states and territories. Recruitment occurs every year in New South Wales, Queensland and Victoria, and every 2 years in the other jurisdictions, timed to coincide with lesbian, gay, bisexual, transgender and queer festivals. Prior to COVID-19, most recruitment (75–80% of the sample) was conducted in person by trained peers who approached potential participants at gay venues and events over the course of a few weeks. Online recruitment occurred afterwards, driven primarily by paid Facebook advertising. The exception is Tasmania, where recruitment is conducted entirely online.

Eligible participants are Australian residents who identify as male (including cisgender and transgender men), aged 16 years or older (online recruitment), 18 years or older (in-person recruitment), who identify as gay, bisexual or queer and/or who have had sex with a man in the past 5 years. Participants are asked to complete a questionnaire about their demographic details, HIV testing and status, use of different HIV prevention methods, sex with casual and regular male partners, relationships, drug use, and sexual health testing and diagnoses. Recall periods are typically the last 6 or 12 months.

For in-person recruitment, trained peers approach potential participants at venues and events and provide information about the study. Consenting participants complete a paper questionnaire (in English) and return it to the recruitment staff. In online recruitment, participants are directed to the study’s website (https://gcpsonline.net), which provides study information and a link to the online questionnaire hosted on the Qualtrics platform (Provo, UT). The online questionnaire uses adaptive routing to exclude ineligible participants and skip irrelevant questions. The online questionnaire is available in English, simplified and traditional Chinese, Indonesian, Portuguese, Spanish, Thai, and Vietnamese. All participants can complete the questionnaire anonymously. Completing the questionnaire is taken as evidence of consent. Participants who complete less than half the study questionnaire are considered to have withdrawn and their responses are not stored. The study has primary institutional ethics approval from the UNSW Sydney Human Research Ethics Committee (ref. HC180903), and the research ethics review panels of the community organisations ACON and Thorne Harbour Health.

### Adjustments to Procedures After COVID-19

COVID-19 restrictions were imposed in Australia after the first two survey rounds of 2020 had been completed (in Victoria and New South Wales), but months before the next scheduled round (in Queensland) was due to take place. In this period, the research team consulted with our community organisation partners and jurisdictional health departments. We determined local restrictions on in-person recruitment (such as bans on large gatherings and mingling in venues), whether recruitment should be delayed or cancelled, and strategies for increasing online recruitment. We revised the procedures for in-person recruitment, including staff wearing masks, maintaining physical distance, limited handling of questionnaire materials, providing hand sanitiser for staff and participants, and using QR codes to direct participants to the online version of the questionnaire. We also added items to the questionnaire to assess the impact of COVID-19.

No scheduled recruitment rounds were cancelled, although some were delayed. In November 2020, in-person recruitment was approved in South Australia (due to eased restrictions), but after recruitment started a short lockdown period was reimposed, and in-person recruitment cancelled. In most jurisdictions, advertising and recruitment were primarily conducted online. Paid Facebook advertising (primarily in English) was used in all jurisdictions, and community organisations advertised the survey through their online networks. In Queensland, New South Wales and Victoria, community organisations placed posters and flyers with QR codes linked to the online questionnaire in venues where in-person recruitment would have taken place. In the early 2021 rounds, our community partners ACON (New South Wales) and Thorne Harbour Health (Victoria) conducted online advertising campaigns on Facebook, and sex and dating apps and websites including Grindr, Hornet, Scruff and Squirt i.e. a greater range of online advertising than in pre-COVID-19 rounds.

### Measures

The survey measures have been previously described [6, 7]. For the current analyses, we included participant characteristics which have been associated with disparities in the Australian HIV epidemic. These included age, region of birth, state of residence, residential suburb, and sexual orientation [10, 11, 14–19]. The proportion of gay men estimated to live in the participant’s residential suburb was calculated using a previously described method [37]. To assess changes in behaviour that may affect likelihood of HIV transmission or timely diagnosis, we include self-reported HIV status, number of recent male sex partners (last 6 months), HIV testing in the last year by non-HIV-positive participants, condom use with casual male partners (last 6 months), PrEP use by HIV-negative participants (last 6 months), and HIV treatment at the time of the survey and viral suppression (last viral load test) by HIV-positive participants. Sex with a risk of HIV transmission was defined as HIV-negative or untested participants who did not use PrEP but reported condomless sex with casual male partners in the 6 months prior to the survey, as defined in earlier publications [6, 9, 10]. Sex between casual partners remains the primary context for HIV transmission among GBM in Australia [38].

Questions added to the survey questionnaire after COVID-19 assessed the impact of the pandemic on income or employment, whether participants had ever been tested for COVID-19, the degree to which participants had been practising physical distancing in the last 6 months, whether COVID-19 had altered the participant’s number of sex partners in the last 6 months, and whether COVID-19 had affected the frequency of PrEP use in the last 6 months (including ‘I wasn’t taking PrEP’, ‘It did not affect my PrEP use’, ‘I took PrEP less often’ and ‘I stopped PrEP’).

### Analyses

We include data collected in July 2017 to June 2021 from five states (New South Wales, Queensland, South Australia, Tasmania and Victoria) which all had recruitment rounds affected by COVID-19. The data were grouped into four 12-month periods to include three pre-COVID-19 and one since COVID-19: July 2017–June 2018, July 2018–June 2019, July 2019–June 2020 (all pre-COVID-19), and July 2020–June 2021 (COVID-19 affected). Sample sizes and sociodemographic characteristics are described, as are the impacts of COVID-19 reported by participants in the 2020–2021 rounds. We report trends in recruitment source (online vs. offline), sociodemographic characteristics, self-reported HIV status of participants, and these key indicators: number of recent male sex partners, HIV testing, HIV treatment, PrEP use, and casual sex with a risk of HIV transmission, as defined above [6, 7]. Bivariate (unadjusted) logistic regression was used to test trends in sociodemographic characteristics for statistical significance, with year as the independent variable and the dependent variable constructed as a binary variable e.g. recruited online vs. offline. The odds ratios produced by these trend analyses indicate the likelihood of participants being in each category (e.g. recruited online) compared with the reference group (e.g. recruited offline) for each additional year in the observed time period (July 2017–June 2021). Trends in key indicators were assessed using multivariate logistic regression, with year as the independent variable, the key indicator as a binary variable (e.g. PrEP use vs. non-use) and demographic variables included to control for variations in sampling. The included control variables were recruitment source (online vs. offline), age (in years), country of birth (Australia vs. overseas), proportion of gay residents in the participant’s suburb (< 10% vs. ≥ 10%), and sexual orientation (gay vs. other). We also assessed whether the trends in key indicators and any COVID-19-related changes had been accentuated by known disparities. This involved stratifying all the key indicators (number of male partners, HIV testing, HIV treatment, PrEP use, and casual sex with a risk of HIV transmission) by age (under 25 vs. 25 years and over), region of birth (Asian born vs. not born in Asia), proportion of gay male residents in the participant’s suburb (< 10% vs. ≥ 10%), and sexual orientation (bisexual vs. not bisexual). Trends in key indicators were then tested with multivariate logistic regression, with time period as the independent variable, the stratification as a covariate (e.g. age as a binary variable) and an interaction term (e.g. time × age) [39]. The odds ratios for the time main effect can be interpreted in a similar way to the trend analyses described above e.g. the odds ratio indicates the likelihood of participants in the stratification group (e.g. under 25 years) reporting the key indicator in question (e.g. More than 10 male partners in the last 6 months) for each additional year in the observed time period. The odds ratio for the stratification main effect (e.g. age) represents the likelihood of participants in the stratification group (e.g. under 25 years) reporting the key indicator in question (e.g. More than 10 male partners in the last 6 months) compared with participants in the reference group (e.g. 25 years and over) irrespective of time (throughout the whole 4 year time period). The interaction term indicates whether the relationship between the stratification groups (e.g. younger vs. older participants) for the key indicator (e.g. number of partners) has changed (widened or lessened) during the 4 year period. Crude and adjusted odds ratios (OR and AOR), 95% confidence intervals (CI) and *p* values are reported. Selected Figures are included, particularly when COVID-19 appears to have accentuated disparities between groups.

## Results

### Sample Size and Participant Characteristics

A total of 31,460 survey responses were included (see Table 1). The majority of participants were recruited from New South Wales (37.1%), Queensland (22.0%) and Victoria (34.4%), the most populous states and the jurisdictions where annual recruitment occurs. Smaller proportions were recruited from South Australia (5.2%) and Tasmania (1.4%), where recruitment occurs every 2 years. In the whole sample, 62.1% were recruited at venues and events, and 37.9% recruited online. The mean age of participants was 37.8 years (SD = 13.5). The majority of participants (70.1%) were born in Australia, and 7.7% were born in Asian countries. Most participants (80.7%) lived in suburbs with fewer than 10% gay male residents, while 19.3% lived in suburbs with ≥ 10% gay male residents. Most participants identified as gay (85.4%), and 9.3% as bisexual. Based on self-reported HIV status, 80.5% of the participants were HIV-negative, 11.1% were untested or did not know their HIV status and 8.4% were HIV-positive.

**Table 1:** Sample characteristics including sample size, proportion recruited online, and sociodemographic characteristics, by recruitment period (pre/post-COVID-19)

	Pre-COVID-19			Post-COVID-19	Trend over time	
	July 2017–June 2018	July 2018–June 2019	July 2019–June 2020	July 2020–June 2021	Odds ratio (95% CI)	*p* value
New South Wales	2860	3167	3337	2293	–	–
Queensland	2079	1826	1772	1250	–	–
South Australia	–	914	–	708	–	–
Tasmania	–	232	–	212	–	–
Victoria	2742	3026	2972	2070	–	–
Recruited at venues and events (%)	6109 (79.5)	6855 (74.8)	6305 (78.0)	259 (4.0)	Ref.	
Recruited online (%)	1572 (20.5)	2310 (25.2)	1776 (22.0)	6274 (96.0)	2.85 (2.77–2.92)	< 0.001
< 25 years old (%)	1162 (15.2)	1586 (17.2)	1088 (13.6)	901 (13.8)	Ref.	
≥ 25 years old (%)	6486 (84.8)	7544 (82.8)	6939 (86.5)	5624 (86.2)	1.07 (1.04–1.10)	< 0.001
Australian born* (%)	5385 (70.4)	6385 (69.8)	5455 (67.7)	4823 (74.0)	1.04 (1.01–1.06)	0.001
Asian born* (%)	558 (7.3)	738 (8.1)	704 (8.7)	436 (6.7)	0.99 (0.95–1.03)	0.627
From suburbs with < 10% gay male residents (%)	6109 (79.9)	7364 (81.4)	6217 (78.2)	5683 (89.3)	Ref.	
From suburbs with ≥ 10% gay male residents (%)	1539 (20.1)	1687 (18.6)	1735 (21.8)	680 (10.7)	0.86 (0.83–0.88)	< 0.001
Gay-identified^a^ (%)	6820 (88.8)	7932 (86.6)	6932 (85.8)	5190 (79.4)	0.80 (0.77–0.82)	< 0.001
Bisexual-identified^a^ (%)	482 (6.3)	767 (8.4)	685 (8.5)	1004 (15.4)	1.37 (1.32–1.42)	< 0.001
HIV-negative^a^ (%)	6138 (79.9)	7503 (81.9)	6624 (82.0)	5058 (77.4)	0.96 (0.93–0.98)	0.002
Untested/unknown status^a^ (%)	860 (11.3)	941 (10.3)	779 (9.6)	904 (13.8)	1.06 (1.03–1.10)	< 0.001
HIV-positive^a^ (%)	675 (8.8)	721 (7.9)	678 (8.4)	571 (8.7)	1.00 (0.97–1.04)	0.808
Total (N)	7681	9165	8081	6533		

*CI* confidence interval

^a^For trend analysis this group was compared with all other participants e.g. Australian born vs. born overseas, gay-identified vs. not gay-identified

Length of residence in Australia was added to the study questionnaire in 2019. In the 2019–2021 rounds, there were 1140 participants who were born in Asia, of whom 157 (13.8%) had lived in Australia for less than 2 years, 270 (23.9%) for 2–5 years and 708 (62.1%) for more than 5 years. Most (n = 655, 57.5%) were in full-time employment and 246 (21.6%) were students.

### Effects of COVID-19 on Recruitment and Sampling

Table 1 shows the sample size for each jurisdiction by year, and trends in sociodemographic characteristics. In all jurisdictions, the sample size was smaller in the COVID-19-affected rounds as most recruitment was switched online (96.0% of participants were recruited online in the COVID-19-affected rounds). The proportion of participants recruited online increased substantially over time (OR 2.85, 95% CI 2.77–2.92, *p* < 0.001). The overall age of the sample increased slightly over time, although the proportion of younger participants (< 25 years old) was similar in the last rounds before COVID-19 (at 13.6% in 2019–2020) and the COVID-19-affected rounds (13.8%). The proportion of Australian born participants increased over time from 70.4% to 74.0% (OR 1.04, 95% CI 1.01–1.06, *p* = 0.001), particularly after COVID-19, while the proportion of Asian born participants remained relatively stable (at 6.7–8.7%; OR 0.99, 95% CI 0.95–1.03, *p* = 0.627). The proportion of participants from suburbs with ≥ 10% gay male residents fell from 20.1 to 10.7% (OR 0.86, 95% CI 0.83–0.88, *p* < 0.001), as did the proportion of gay-identified participants (from 88.8 to 79.4%; OR 0.80, 95% CI 0.77–0.82, *p* < 0.001), with the greatest year on year change occurring after COVID-19 emerged. The proportion of participants who were HIV-negative fell slightly in the COVID-19-affected rounds (to 77.4%; OR 0.96, 95% CI 0.93–0.98, *p* = 0.002), while the proportion who were untested or did not know their HIV status increased to 13.8% (OR 1.06, 95% CI 1.03–1.10, *p* < 0.001). The proportion of participants who were HIV-positive remained stable at 7.9–8.8% (OR 1.00, 95% CI 0.97–1.04, *p* = 0.808).

### Impacts of COVID-19 on Participants

In the COVID-19-affected rounds (2020–2021), 6533 surveys were completed. Of these participants, 26.9% reported that they had lost income or their job because of COVID-19, 51.9% had been tested for COVID-19, and 88.1% had been practising physical distancing (staying at home or away from other people) in the previous 6 months. Over half the participants (52.1%) reported that they had had fewer sex partners in the 6 months prior to the survey because of COVID-19. Among former and current PrEP users (n = 1702), 26.3% reported that they had taken PrEP less often in the last 6 months and 11.9% had stopped using PrEP because of COVID-19.

### Trends in Key Indicators

Table 2 shows trends in key HIV-related indicators, with adjustments for variations in sampling. The proportion of all participants reporting more than 10 male sex partners in the 6 months prior to survey was 21.2–23.1% during 2017–2020 and fell to 15.6% after COVID-19 emerged. The adjusted trend analysis indicated the long-term trend in partner numbers was stable (AOR 0.99, 95% CI 0.95–1.02, *p* = 0.504). Similarly, the proportion of participants reporting recent HIV testing (in the year prior to the survey) fell in the COVID-19-affected rounds (from 67.8–69.2% in 2017–2020 to 56.6% in 2020–2021), but the adjusted trend analysis indicated that the long-term trend in testing was upwards (AOR 1.04, 95% CI 1.01–1.07, *p* = 0.014). The proportion of HIV-positive men on HIV treatment remained stable in both crude and adjusted trends (at 92.9–96.2%; AOR 1.19, 95% CI 0.94–1.50, *p* = 0.143), as did the proportion of HIV-positive participants on treatment who reported an undetectable viral load (94.1–97.7%; not shown in Table 2). PrEP use increased over time (reaching 36.0% in 2019–2020) but fell in the COVID-19-affected rounds to 29.3%, although the long-term trend remained upward (AOR 1.39, 95% CI 1.34–1.44, *p* < 0.001). The long-term trend in the proportion of participants with casual male partners who were classified as at risk of HIV infection fell over time (AOR 0.89, 95% CI 0.86–0.93, *p* < 0.001) reaching 21.0% in 2019–2020, but increased in the rounds after COVID-19 emerged to 26.8%. However, this was in the context of participants reporting fewer male partners overall and less casual sex in the COVID-19-affected rounds. For example, in the 2019–2020 rounds 62.4% of participants (5046/8081) reported any sex with casual male partners while in the 2020–2021 this proportion fell to 55.9% (3655/6533).

**Table 2:** Trends in key indicators

	Pre-COVID-19			Post-COVID-19	Trend over time			
	July 2017–June 2018	July 2018–June 2019	July 2019–June 2020	July 2020–June 2021	Crude odds ratio (95% CI)	*p* value	Adjusted odds ratio^a^ (95% CI)	*p* value
More than 10 male partners in the last 6 months (%)	1771 (23.1)	1946 (21.2)	1774 (22.0)	1022 (15.6)	0.88 (0.86–0.90)	< 0.001	0.99 (0.95–1.02)	0.504
Total (all participants)	7681	9165	8081	6533				
HIV testing in the last 12 months (%)	4748 (67.8)	5813 (68.8)	5126 (69.2)	3375 (56.6)	0.87 (0.85–0.90)	< 0.001	1.04 (1.01–1.07)	0.014
Total (non-HIV-positive men)^b^	7006	8444	7403	5962				
On HIV treatment at the time of the survey (%)	620 (95.0)	568 (95.6)	600 (92.9)	536 (96.2)	1.01 (0.86–1.19)	0.874	1.19 (0.94–1.50)	0.143
Total (HIV-positive men)	653	594	678	557				
PrEP use in the last 6 months (%)	1299 (22.1)	2108 (28.2)	2495 (36.0)	1702 (29.3)	1.16 (1.13–1.19)	< 0.001	1.39 (1.34–1.44)	< 0.001
Total (non-HIV-positive men^b^	5882	7486	6925	5811				
HIV-negative and untested/unknown status participants who did not use PrEP and had condomless sex with casual male partners in the last 6 months i.e. at risk of HIV infection (%)	1385 (28.7)	1435 (25.7)	1061 (21.0)	980 (26.8)	0.93 (0.91–0.96)	< 0.001	0.89 (0.86–0.93)	< 0.001
Total (participants with casual male partners in the last 6 months)	4824	5575	5046	3655				

*CI* confidence interval, *PrEP* pre-exposure prophylaxis

^a^Adjusted for the following variations in sampling: recruitment source, age, country of birth, proportion of gay residents in participant’s suburb, and sexual orientation

^b^Denominators vary due to exclusion of participants with missing data

### Assessing Disparities in COVID-19-Related Changes

We assessed whether changes in key indicators, particularly in the COVID-19-affected rounds, were accentuated by disparities previously identified in the Australian HIV epidemic. These stratifications are shown in Table 3.

**Table 3:** Trends in key indicators over time, showing stratifications and interaction effects

	Pre-COVID-19			Post-COVID-19	Stratification main effect		Time main effect (trend over time)		Interaction effect (time × stratification)	
	July 2017–June 2018	July 2018–June 2019	July 2019–June 2020	July 2020–June 2021	Adjusted odds ratio (95% CI)	*p* value	Adjusted odds ratio (95% CI)	*p* value	Adjusted odds ratio (95% CI)	*p* value
More than 10 male partners in the last 6 months										
< 25 years old (%)	194 (16.7)	233 (14.9)	164 (15.1)	85 (9.4)	0.65 (0.53–0.80)	< 0.001	0.88 (0.86–0.90)	< 0.001	0.96 (0.88–1.04)	0.317
≥ 25 years old (%)	1571 (24.2)	1701 (22.6)	1587 (22.9)	932 (16.6)	Ref		Ref		Ref	
Asian born (%)	121 (21.7)	130 (17.6)	146 (20.7)	64 (14.7)	1.03 (0.93–1.14)	0.167	0.88 (0.86–0.90)	< 0.001	0.36 (0.33–0.38)	0.605
Not born in Asia (%)	1650 (23.2)	1816 (21.6)	1628 (22.1)	958 (15.7)	Ref		Ref		Ref	
From suburbs with < 10% gay male residents (%)	1320 (21.6)	1439 (19.5)	1269 (20.4)	815 (14.3)	0.76 (0.64–0.89)	0.001	0.96 (0.91–1.02)	0.204	0.91 (0.85–0.97)	0.004
From suburbs with ≥ 10% gay male residents (%)	445 (28.9)	486 (28.8)	484 (27.9)	179 (26.3)	Ref		Ref		Ref	
Bisexual-identified (%)	90 (18.7)	114 (14.9)	95 (13.9)	107 (10.7)	0.79 (0.60–1.04)	0.087	0.90 (0.87–0.92)	< 0.001	0.91 (0.82–1.00)	0.056
Not bisexual-identified (%)	1681 (23.4)	1832 (21.8)	1679 (22.7)	915 (16.6)	Ref		Ref		Ref	
Total (all participants)	7681	9165	8081	6533						
HIV testing in the last 12 months										
< 25 years old (%)	726 (63.5)	972 (63.2)	646 (61.1)	399 (44.7)	0.89 (0.76–1.05)	0.170	0.88 (0.86–0.90)	< 0.001	0.90 (0.85–0.96)	< 0.001
≥ 25 years old (%)	4007 (68.7)	4813 (70.2)	4450 (70.6)	2975 (58.8)	Ref		Ref		Ref	
Asian born (%)	354 (68.9)	517 (74.5)	491 (74.1)	235 (58.3)	1.19 (0.93–1.52)	0.162	0.87 (0.85–0.89)	< 0.001	1.01 (0.92–1.11)	0.802
Not born in Asia (%)	4394 (67.7)	5296 (68.3)	4635 (68.8)	3140 (56.5)	Ref		Ref		Ref	
From suburbs with < 10% gay male residents (%)	3700 (66.1)	4590 (67.3)	3838 (67.2)	2874 (55.2)	0.81 (0.68–0.97)	0.019	1.00 (0.94–1.07)	0.867	0.86 (0.81–0.93)	< 0.001
From suburbs with ≥ 10% gay male residents (%)	1034 (75.0)	1172 (76.9)	1217 (77.3)	437 (73.7)	Ref		Ref		Ref	
Bisexual-identified (%)	291 (63.7)	420 (56.8)	375 (57.5)	438 (45.1)	0.84 (0.67–1.04)	0.104	0.90 (0.87–0.92)	< 0.001	0.88 (0.82–0.95)	0.001
Not bisexual-identified (%)	4457 (68.1)	5393 (70.0)	4751 (70.4)	2937 (58.9)	Ref		Ref		Ref	
Total (non-HIV-positive men)	7006	8444	7403	5962						
On HIV treatment at the time of the survey										
< 25 years old (%)	15 (88.2)	18 (75.0)	19 (76.0)	4 (57.1)	0.53 (0.10–2.79)	0.451	1.08 (0.91–1.3)	0.369	0.59 (0.32–1.11)	0.104
≥ 25 years old (%)	601 (95.1)	545 (96.5)	577 (94.1)	531 (97.1)	Ref		Ref		Ref	
Asian born (%)	37 (90.2)	33 (91.7)	32 (86.5)	29 (96.7)	0.37 (0.09–1.47)	0.158	1.00 (0.84–1.18)	0.966	1.16 (0.67–2.01)	0.599
Not born in Asia (%)	583 (95.3)	535 (95.9)	568 (93.3)	507 (96.2)	Ref		Ref		Ref	
From suburbs with < 10% gay male residents (%)	470 (94.6)	441 (95.0)	447 (91.6)	450 (97.4)	0.50 (0.13–1.84)	0.295	1.10 (0.67–1.81)	0.704	1.00 (0.59–1.69)	0.989
From suburbs with ≥ 10% gay male residents (%)	148 (96.1)	117 (98.3)	146 (98.0)	81 (96.4)	Ref		Ref		Ref	
Bisexual-identified (%)	19 (82.6)	21 (87.5)	25 (89.3)	27 (84.4)	0.29 (0.07–1.24)	0.095	1.02 (0.86–1.22)	0.781	1.02 (0.61–1.70)	0.948
Not bisexual-identified (%)	601 (95.4)	547 (96.0)	575 (93.0)	509 (97.0)	Ref		Ref		Ref	
Total (HIV-positive men)	653	594	678	557						
PrEP use in the last 6 months										
< 25 years old (%)	137 (14.5)	211 (15.6)	225 (22.9)	140 (16.3)	0.52 (0.42–0.65)	< 0.001	1.16 (1.13–1.19)	< 0.001	0.95 (0.88–1.03)	0.229
≥ 25 years old (%)	1159 (23.6)	1887 (31.0)	2257 (38.2)	1561 (31.5)	Ref		Ref		Ref	
Asian born (%)	83 (21.1)	155 (26.4)	212 (34.3)	121 (30.8)	0.86 (0.65–1.13)	0.285	1.16 (1.13–1.19)	< 0.001	1.05 (0.95–1.16)	0.344
Not born in Asia (%)	1216 (22.2)	1953 (28.3)	2283 (36.2)	1581 (29.2)	Ref		Ref		Ref	
From suburbs with < 10% gay male residents (%)	928 (19.8)	1527 (25.3)	1764 (33.2)	1379 (27.2)	0.88 (0.83–0.94)	< 0.001	0.64 (0.54–0.76)	< 0.001	0.38 (0.33–0.44)	< 0.001
From suburbs with ≥ 10% gay male residents (%)	371 (31.6)	571 (41.9)	708 (46.8)	303 (51.4)	Ref		Ref		Ref	
Bisexual-identified (%)	52 (14.1)	98 (15.5)	123 (20.8)	168 (18.2)	0.56 (0.41–0.76)	< 0.001	1.19 (1.16–1.22)	< 0.001	0.93 (0.84–1.03)	0.176
Not bisexual-identified (%)	1247 (22.6)	2010 (29.3)	2372 (37.5)	1534 (31.4)	Ref		Ref		Ref	
Total (non-HIV-positive men)	5882	7486	6925	5811						
HIV-negative and untested/unknown status participants who did not use PrEP and had condomless sex with casual male partners in the last 6 months i.e. at risk of HIV infection										
< 25 years old (%)	288 (40.9)	345 (38.8)	204 (33.3)	162 (39.2)	2.02 (1.64–2.49)	< 0.001	0.94 (0.91–0.97)	0.001	1.00 (0.92–1.09)	0.962
≥ 25 years old (%)	1091 (26.6)	1078 (23.2)	842 (19.1)	815 (25.2)	Ref		Ref		Ref	
Asian born (%)	86 (25.0)	109 (24.3)	103 (23.2)	56 (22.0)	0.88 (0.64–1.2)	0.413	0.93 (0.9–0.96)	< 0.001	1.01 (0.9–1.14)	0.825
Not born in Asia (%)	1299 (29.0)	1326 (25.9)	958 (20.8)	924 (27.2)	Ref		Ref		Ref	
From suburbs with < 10% gay male residents (%)	1130 (29.9)	1199 (27.1)	859 (22.6)	874 (27.9)	1.15 (0.93–1.43)	0.205	0.83 (0.76–0.9)	< 0.001	1.14 (1.04–1.24)	0.005
From suburbs with ≥ 10% gay male residents (%)	250 (24.3)	218 (19.9)	187 (15.8)	78 (17.9)	Ref		Ref		Ref	
Bisexual-identified (%)	81 (27.2)	143 (31.5)	105 (26.7)	200 (36.4)	0.78 (0.58–1.05)	0.100	0.90 (0.87–0.93)	< 0.001	1.25 (1.13–1.38)	< 0.001
Not bisexual-identified (%)	1304 (28.8)	1292 (25.2)	956 (20.6)	780 (25.1)	Ref		Ref		Ref	
Total (participants with casual male partners in the last 6 months)	4824	5575	5046	3655						

*CI* confidence interval, *PrEP* pre-exposure prophylaxis

**Fig. 1:**
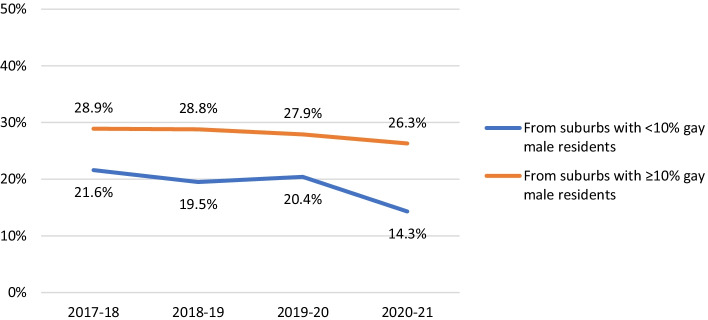
Proportion of all participants reporting more than 10 male partners in the last 6 months, by suburb

Younger men (aged < 25 years) and participants from suburbs with fewer (< 10%) gay male residents were consistently less likely than their respective comparison groups (older men and participants from suburbs with ≥ 10% gay male residents) to report more than 10 recent male sex partners (age main effect AOR 0.65, 95% CI 0.53–0.80, *p* < 0.001; suburb main effect AOR 0.76, 95% CI 0.64–0.89, *p* = 0.001). Partner numbers declined over time (2017–21) among younger men (AOR 0.88, 95% CI 0.86–0.90, *p* < 0.001), Asian born participants (AOR 0.88, 95% CI 0.86–0.90, *p* < 0.001), and bisexual men (AOR 0.90, 95% CI 0.87–0.92, *p* < 0.001). After COVID-19 emerged, partner numbers declined more noticeably among participants from suburbs with fewer gay male residents (interaction AOR 0.91, 95% CI 0.85–0.97, *p* = 0.004), compared with participants from suburbs with more gay male residents, among whom partner numbers largely remained stable (see Fig. 1).

**Fig. 2:**
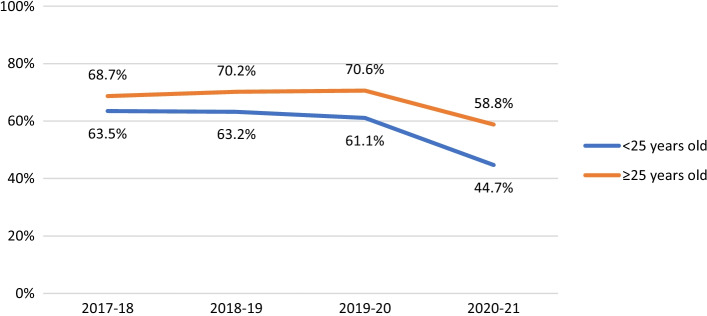
Proportion of non-HIV-positive participants reporting HIV testing in the last 12 months, by age group

**Fig. 3:**
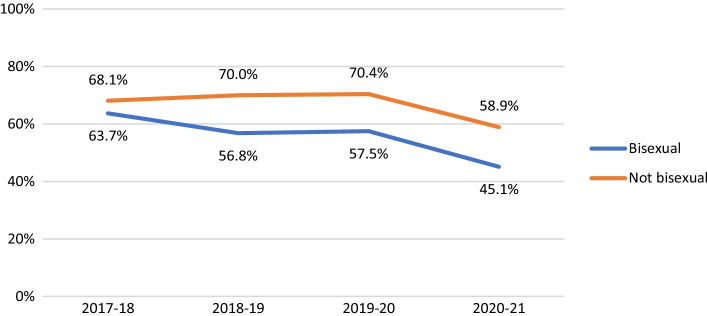
Proportion of non-HIV-positive participants reporting HIV testing in the last 12 months, by sexual orientation

Among non-HIV-positive participants, HIV testing in the last year was consistently less likely to be reported by participants in suburbs with fewer gay male residents (AOR 0.81, 95% CI 0.68–0.97, *p* = 0.019). HIV testing fell over time among younger men (see Fig. 2; AOR 0.88, 95% CI 0.86–0.90, *p* < 0.001), Asian born participants (AOR 0.87, 95% CI 0.85–0.89, *p* < 0.001), and bisexual men (see Fig. 3; AOR 0.90, 95% CI 0.87–0.92, *p* < 0.001). There were steeper declines in recent HIV testing after COVID-19 emerged in younger men (Fig. 2; interaction AOR 0.90, 95% CI 0.85–0.96, *p* < 0.001), participants in suburbs with fewer gay male residents (interaction AOR 0.86, 95% CI 0.81–0.93, *p* < 0.001) and bisexual men (Fig. 3; interaction AOR 0.88, 95% CI 0.82–0.95, *p* = 0.001) than in their comparison groups, creating wider differences in levels of HIV testing.

HIV-positive participants appeared to be less likely to report being on HIV treatment if they were aged under 25 years old or bisexual, but these were not statistically independent differences in the multivariate regression models (e.g. age main effect AOR 0.53, 95% CI 0.10–2.79, *p* = 0.451; sexual orientation main effect AOR 0.29, 95% CI 0.07–1.24, *p* = 0.095), nor did treatment levels change in these groups over time (age trend AOR 1.08, 95% CI 0.91–1.30, *p* = 0.369; sexual orientation trend AOR 1.02, 95% CI 0.86–1.22, *p* = 0.781). Asian born HIV-positive participants were as likely as participants born elsewhere to report being on HIV treatment (AOR 0.37, 95% CI 0.09–1.47, *p* = 0.158), and this did not change over time (AOR 1.00, 95% CI 0.84–1.18, *p* = 0.966). HIV-positive participants from suburbs with fewer gay residents appeared to be slightly less likely to report being on treatment than participants from suburbs with more gay residents, but this difference was not statistically independent (AOR 0.50, 95% CI 0.13–1.84, *p* = 0.295), and treatment levels did not change over time in this group (AOR 1.10, 95% CI 0.67–1.81, *p* = 0.704).

**Fig. 4:**
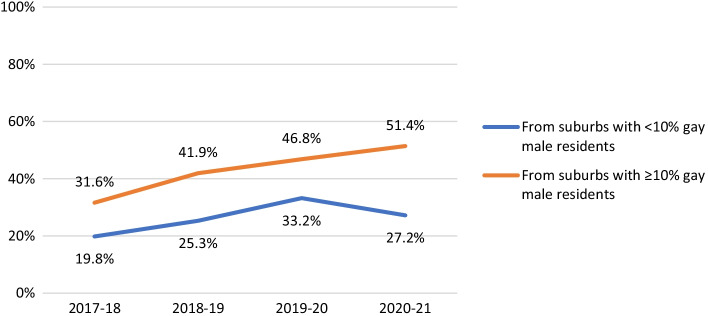
Proportion of non-HIV-positive participants who reported PrEP use in the last 6 months, by suburb

Among non-HIV-positive participants, PrEP use was consistently lower among men aged under 25 years old (AOR 0.52, 95% CI 0.42–0.65, *p* < 0.001), among participants from suburbs with fewer gay male residents (see Fig. 4; AOR 0.88, 95% CI 0.83–0.94, *p* < 0.001), and bisexual men (AOR 0.56, 95% CI 0.41–0.76, *p* < 0.001). PrEP use was reported at similar levels by Asian born participants and participants born elsewhere (AOR 0.86, 95% CI 0.65–1.13, *p* = 0.285). PrEP use increased over time among younger men (AOR 1.16, 95% CI 1.13–1.19, *p* < 0.001), Asian born participants (AOR 1.16, 95% CI 1.13–1.19, *p* < 0.001) and bisexual men (AOR 1.19, 95% CI 1.16–1.22, *p* < 0.001), but in all cases fell after COVID-19. After COVID-19 emerged, PrEP use fell in suburbs with fewer gay male residents, but it continued to increase in suburbs with more gay male residents, so the difference in levels of use between the two groups widened (see Fig. 4; interaction AOR 0.38, 95% CI 0.33–0.44, *p* < 0.001). After COVID-19, PrEP use by bisexual men decreased, but it decreased more among non-bisexual participants, so the difference in levels of use between the two groups narrowed; this was not a statistically independent effect (interaction AOR 0.93, 95% CI 0.84–1.03, *p* = 0.176).

**Fig. 5:**
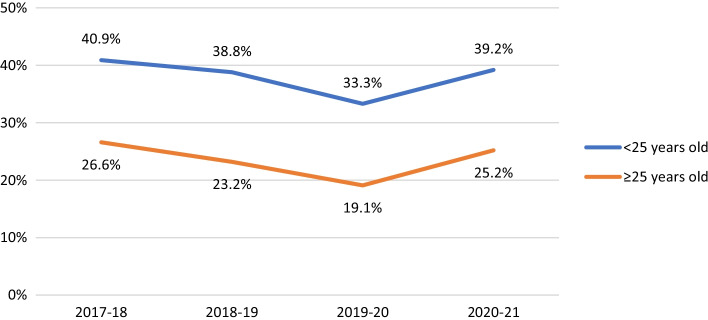
Proportion of non-HIV-positive participants who reported casual sex with a risk of HIV infection in the last 6 months, by age group

**Fig. 6:**
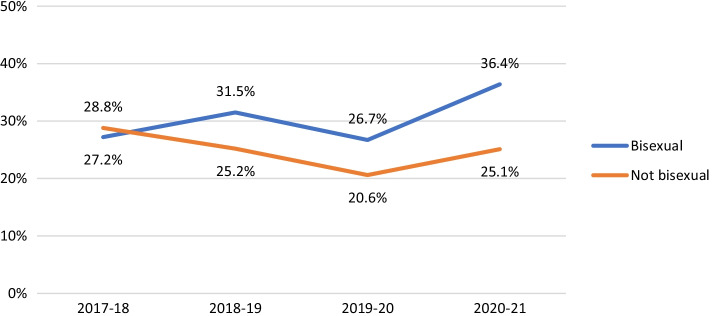
Proportion of non-HIV-positive participants who reported casual sex with a risk of HIV infection in the last 6 months, by sexual orientation

Non-HIV-positive participants under the age of 25 were consistently more likely than older participants to report condomless sex with casual partners while not using PrEP (i.e. sex with a risk of HIV transmission; AOR 2.02, 95% CI 1.64–2.49, *p* < 0.001). Younger men became slightly less likely to report risk over time (AOR 0.94, 95% CI 0.91–0.97, *p* < 0.001) although the gap between younger and older participants in reported levels of risk did not change after COVID-19 emerged (see Fig. 5; interaction AOR 1.00, 95% CI 0.92–1.09, *p* = 0.962). Asian born participants were as likely as other participants to report casual sex with a risk of HIV transmission (AOR 0.88, 95% CI 0.64–1.20, *p* = 0.413), and became less likely to report risk over time (AOR 0.93, 95% CI 0.90–0.96, *p* < 0.001). Participants from suburbs with < 10% gay male residents were as likely as other participants to report sex with a risk of HIV transmission (AOR 1.15, 95% CI 0.93–1.43, *p* = 0.205), and had become less likely to report risk over time (AOR 0.83, 95% CI 0.76–0.90, *p* < 0.001). However, after COVID-19 emerged, casual sex with a risk of infection increased more in suburbs with fewer gay male residents than in suburbs with ≥ 10% gay male residents, widening the difference in levels of risk between the two groups (interaction AOR 1.14, 95% CI 1.04–1.24, *p* < 0.005). Overall, bisexual men were as likely as other participants to report casual sex with a risk of transmission (AOR 0.78, 95% CI 0.58–1.05, *p* = 0.100), and had become less likely to report risk over time (AOR 0.90, 95% CI 0.87–0.93, *p* < 0.001). However, after COVID-19 emerged, the gap between the two groups widened, with bisexual men being more likely to report risk in the COVID-19-affected rounds (see Fig. 6; interaction AOR 1.25, 95% CI 1.13–1.38, *p* < 0.001).

## Discussion

COVID-19 forced us to adapt behavioural HIV surveillance of GBM in Australia to maintain the timely supply of relevant data to guide HIV and sexual health programs. With support from our community partners and health departments, we successfully adjusted recruitment procedures in participating jurisdictions. The increased reliance on online recruitment changed the sample profile, broadening the geographic spread of the sample and increasing the participation of bisexual men, as has been noted in other studies [5]. Participants reported a range of impacts of COVID-19, with most limiting contact with other people, and over half the sample reducing the amount of sex they had. Over a quarter of PrEP users reduced how often they were taking PrEP, and one in ten stopped taking PrEP because of COVID-19. These findings are broadly consistent with other studies conducted in Australia and overseas on the impact of COVID-19 on GBM’s behaviour [21, 23, 25–32, 40]. As the largest regular surveys of GBM conducted in Australia, our results are likely to provide some of the best estimates of how COVID-19 has affected GBM’s behaviour at a community level.

We assessed the impact of COVID-19 on key behavioural indicators related to the Australian HIV epidemic. The number of male sex partners, recent HIV testing and PrEP use all fell substantially after COVID-19 emerged, although longer-term trends in these indicators appeared unaffected after adjusting for variations in sampling. HIV treatment and viral suppression were sustained at high levels, and did not seem to be affected by COVID-19, reflecting the quick response of HIV-positive people and clinical services to sustain access to treatment, such as using telehealth consultations [41]. Although the overall proportion of GBM reporting casual sex fell after COVID-19, among those who continued to have casual sex, the proportion reporting condomless sex with a risk of HIV transmission increased, linked to the drop in PrEP coverage. Overall, these changes paint a mixed picture of the impact of COVID-19 on GBM and the HIV epidemic in Australia, with less sex and risk of HIV, but also less use of HIV testing and protection from condoms or PrEP. As others have noted [22, 32], encouraging GBM to reengage with testing services and maintain effective prevention methods is critical, particularly if they continued to have (casual) sex throughout the COVID-19 pandemic, or if they are returning to pre-pandemic levels of sexual activity.

We assessed whether changes in GBM’s behaviour prompted by COVID-19 had been accentuated by known disparities in the Australian epidemic [10, 11, 14–19]. Younger GBM (under the age of 25) have been previously found to report fewer sex partners than older men but, if they have casual sex, to be as likely as older peers to report condomless sex [18]. Younger men have also been found to be less likely to engage in HIV testing or use PrEP [14, 18], and to remain at higher risk of HIV infection, even if they have access to PrEP [13]. Our analysis reinforces these findings. Although younger participants reported a bigger decline in partner numbers after COVID-19, they also had greater reductions in HIV testing and PrEP use than older participants. Younger men who reported casual sex were consistently more likely than older participants to report condomless sex with a risk of HIV transmission, and this did not change after COVID-19. Due to recent declines in HIV testing and PrEP use, this suggests that younger GBM should be a particular focus of HIV prevention efforts in the COVID-19 period.

In contrast to Australian-born GBM, among whom annual HIV diagnoses have fallen in the last few years, HIV diagnoses among Asian-born GBM have been increasing in Australia [15–17, 42]. Asian GBM diagnosed with HIV in Melbourne and Sydney between 2014 and 2018 reported fewer sex partners than Australian-born men but were less likely to have previously tested for HIV or have access to public health insurance (Medicare) [17]. However, Asian participants in our sample reported similar behaviour to other participants in terms of partner numbers, HIV testing, PrEP use and risk of HIV, and COVID-19 did not create any obvious disparities between Asian-born and other participants. This may reflect the fact that most Asian-born participants in the Gay Community Periodic Surveys had been living in Australia for over 5 years, were likely to be well integrated into community networks, and have similar access to HIV testing and prevention as Australian-born GBM. Border restrictions imposed after COVID-19 may also have suppressed the number of recently arrived Asian-born GBM in Australia, particularly in the 2020–2021 rounds.

The geographic distribution of gay men (and HIV) in Australia is highly variable, with most postcodes estimated to have few gay residents and very low HIV prevalence, and a small number of mainly inner-city areas with larger gay populations and more people living with HIV [37, 43]. In the initial period of PrEP rollout in New South Wales, uptake was faster and there were greater reductions in HIV incidence in suburbs with more gay residents [11, 13]. Our results confirm that the proportion of gay residents may be important for HIV-related behaviours and outcomes across the country [44]. Compared with participants from suburbs with more gay residents, participants from suburbs with fewer (< 10%) gay residents (the majority of the sample) reported fewer partners, less HIV testing and PrEP use, but also more risk for HIV when having casual sex. All of these disparities increased after COVID-19 emerged.

It has been previously found that bisexual men in Australia generally have fewer male partners than gay men, are much less likely to have ever been or recently tested for HIV, and use condoms more consistently during anal sex [19, 45]. Previous studies in Australia have not found differences between bisexual and gay men in levels of PrEP knowledge and use, although relatively few bisexual men participated in these studies [46–48]. A recent analysis suggests bisexual men have become overrepresented among GBM at risk of HIV, after many gay men have adopted PrEP use [10]. Our results reaffirm and extend these findings, showing that bisexual men consistently report fewer male partners, are less likely to be tested for HIV or use PrEP than gay men and other participants, but if they have casual sex with male partners, they are more likely to report sex with a risk of HIV transmission. After COVID-19, partner numbers and HIV testing fell more among bisexual men than other participants, and the risk of HIV for bisexual men having casual sex with male partners increased. PrEP use fell less among bisexual men than other participants after COVID-19, but remained much lower than among gay and other identified men.

Although HIV treatment did not appear to be affected by COVID-19 in our sample, we note that treatment levels appeared to be consistently lower among HIV-positive participants aged under 25 and bisexual men. These were not statistically independent differences, probably due to the relatively small sample sizes of these groups. Low levels of HIV treatment in younger GBM living with HIV were found over 10 years ago, suggesting treatment uptake remains an issue in this group [49]. Others have noted a lack of research about younger GBM living with HIV in Australia and the challenges they may face initiating or sustaining treatment [50, 51], particularly if they do not have access to Medicare [15], and we recommend this be investigated further. Other Australian research has not found a difference in HIV treatment use between bisexual and gay men, but showed that bisexual men living with HIV may experience more stigma and lack social support compared with gay men living with HIV [52].

Previous international reviews of behavioural surveillance studies have recommended that studies should have a backup sampling method [8]. We were fortunate we had the option of switching between in-person recruitment to online advertising after COVID-19, as these methods were already incorporated into the protocol for the Gay Community Periodic Surveys. However, relying on online recruitment meant that, although the range of participants we recruited was extended (e.g. geography, sexual orientation), our overall sample size decreased, potentially limiting the depth of the information we collected and the analyses we could perform. The Australian behavioural surveillance system is unusual in having sustained venue- and event-based recruitment of GBM at scale for 25 years. We have previously found that even the largest online surveys of GBM in Australia cannot reproduce the sample sizes achieved with face-to-face recruitment [45, 53], in contrast to other countries [8]. Although in person recruitment is labour intensive and relatively expensive (involving teams of paid staff to undertake recruitment), in Australia it is often quicker than online recruitment of GBM, and achieves a larger sample. A hybrid approach involving both face-to-face and online recruitment is likely to remain strategic in Australia, to maintain continuity over time but also extend reach and allow flexibility in responding to adverse events like COVID-19.

The Gay Community Periodic Surveys are consistent with guidelines for behavioural surveillance in allowing participants to take part anonymously, encouraging honesty [3, 4], but this is a known limitation, as we cannot assess the responses of individuals over time. Also consistent with international guidelines, the surveys target GBM who may be at risk of HIV. This means that the samples we recruit are not representative of all GBM in Australia, which would likely feature a wider age range, more bisexual men and more participants from regional and rural Australia [54]. Given the evolution of the Australian HIV epidemic, and the overrepresentation of Asian-born GBM in annual diagnoses [15, 16, 42], an additional problem is whether we recruit sufficient numbers of recently arrived Asian-born GBM to describe their practices and identify points of intervention. That is something we plan to address in future. Finally, we acknowledge that the changes we made to recruitment processes in response to COVID-19 (e.g. emphasising online advertising) changed the participant profile, and may have excluded those without reliable access to the internet [2]. It is therefore likely that some of the behavioural changes we observed were not solely due to COVID-19 but due to changes in sampling, although we did adjust our trend analyses for changes in the sample profile.

Our analysis of behavioural surveillance data collected from five states across Australia shows the varied impact of COVID-19 on GBM’s behaviour. Most GBM reduced their levels of sexual activity and risk of HIV during COVID-19, but some subgroups appeared to be at increased risk after COVID-19, if they continued or returned to having casual sex with male partners. These included GBM under the age of 25, those living in suburbs with fewer gay residents, and bisexual men. These groups should be prioritised when encouraging GBM to reengage with HIV testing services and effective prevention methods, like condoms and PrEP.

## Data Availability

A copy of the dataset used for this analysis will be provided to bona fide researchers on request.

## Code Availability (Software Application or Custom Code)

A copy of the syntax used in this analysis will be provided on request.
